# Mitoxantrone-Loaded Nanoferritin Slows Tumor Growth and Improves the Overall Survival Rate in a Subcutaneous Pancreatic Cancer Mouse Model

**DOI:** 10.3390/biomedicines9111622

**Published:** 2021-11-05

**Authors:** Giamaica Conti, Martina Pitea, Riccardo Ossanna, Roberta Opri, Giada Tisci, Elisabetta Falvo, Giulio Innamorati, Esther Ghanem, Andrea Sbarbati, Pierpaolo Ceci, Giulio Fracasso

**Affiliations:** 1 Department of Neurological and Movement Sciences, University of Verona, 37134 Verona, Italy; giamaica.conti@univr.it (G.C.); riccardo.ossanna@univr.it (R.O.); andrea.sbarbati@univr.it (A.S.); 2 Department of Biochemical Sciences, University Sapienza, 00185 Rome, Italy; martina.pitea@uniroma1.it (M.P.); tisci.1887341@studenti.uniroma1.it (G.T.); 3 Center for Life Nano Science@Sapienza Istituto Italiano di Tecnologia, 00161 Rome, Italy; 4 Department of Medicine, University of Verona, 37134 Verona, Italy; roberta.opri@gmail.com; 5 Institute of Molecular Biology and Pathology, CNR—National Research Council of Italy, 00185 Rome, Italy; elisabetta.falvo@cnr.it; 6 Department of Surgical Sciences, Dentistry, Gynecology and Pediatrics, Section of Surgery, University of Verona, 37134 Verona, Italy; giulio.innamorati@univr.it; 7 Department of Sciences, Notre Dame University-Louaize, Zouk Mosbeh P.O. Box 72, Lebanon; eghanem@ndu.edu.lb

**Keywords:** targeted therapy, pancreatic cancer, human ferritin, transferrin receptor (CD71), mitoxantrone

## Abstract

Pancreatic cancer (PC) represents an intriguing topic for researchers. To date, the prognosis of metastasized PC is poor with just 7% of patients exceeding a five-year survival period. Thus, molecular modifications of existing drugs should be developed to change the course of the disease. Our previously generated nanocages of Mitoxantrone (MIT) encapsulated in human H-chain Ferritin (HFt), designated as HFt-MP-PASE-MIT, has shown excellent tumor distribution and extended serum half-life meriting further investigation for PC treatment. Thus, in this study, we used the same nano-formulation to test its cytotoxicity using both in vitro and in vivo assays. Interestingly, both encapsulated and free-MIT drugs demonstrated similar killing capabilities on PaCa44 cell line. Conversely, in vivo assessment in a subcutaneous PaCa44 tumor model of PC demonstrated a remarkable capability for encapsulated MIT to control tumor growth and improve mouse survival with a median survival rate of 65 vs. 33 days for loaded and free-MIT, respectively. Interestingly, throughout the course of mice treatment, MIT encapsulation did not present any adverse side effects as confirmed by histological analysis of various murine tissue organs and body mass weights. Our results are promising and pave the way to effective PC targeted chemotherapy using our HFt nanodelivery platforms.

## 1. Introduction

Pancreatic cancer (PC) is a life-threatening disease with cases still on the rise, especially in Western countries, despite hundreds of research studies and transdisciplinary theranostic approaches. The prevalence of PC was 458,918 in 2018 with a projected trend of increase to 639,030 in 2030 [1,2]. Actually, PC is the seventh cause of tumor mortality, but it is predicted to become the second leading cause of cancer death. PC is a sneaky disease since some patients do not experience any symptoms during the initial stages of tumor growth. When the tumor is large enough to cause symptoms, it has already spread beyond the organ and this late diagnosis contributes to a very poor prognosis with a five-year survival rate (i.e., below 5%) [3].

Nowadays, surgery remains the only therapeutic intervention with a curative aim, but unfortunately less than 15–20% of patients are eligible for surgery showing a resectable cancer [4]. Nevertheless, only 20% of this group can survive up to five years due to rigorous complications and relapses [4]. Neo-adjuvant and adjuvant therapies based on chemotherapy, combined-chemotherapy, or chemo-radiotherapy have failed their curative intent while decreasing relapses. Furthermore, these treatments showed only palliative effects when applied in the management of metastatic disease. Therefore, the limitations of the current therapeutic approaches reinforce the need to explore new ways to advance the clinical outcomes.

Many tumor-associated antigens (TAAs) have been investigated in the last ten years as targeted biomarkers to improve the intoxication of PC cells and therefore to control or completely cure this malignancy. Among the more studied and evaluated TAAs in clinical trials are mucin 1 (MUC1), prostate stem cell antigen (PSCA), carcinoembryonic antigen (CEA) and mesothelin. In particular, mesothelin has gained attention in the last years as it is highly expressed in PC tumors reaching 80% levels, with undetectable expression in healthy tissues. However, so far, mesothelin-based therapies (i.e., as antibodies, antibody drug conjugates, vaccines and CAR-T cells) have not significantly changed the outcome in PC treatment [5].

On the other hand, CD71, also known as transferrin receptor 1 (TfR1), shows outperforming properties as it is overexpressed in many hematological and solid tumors, accessible extracellularly and internalized. Taken together, CD71 is an attractive portal for drug delivery. However, traces of CD71 expression have been detected in healthy proliferating tissues, increasing the vulnerability of normal cells to CD71-based therapies. Therefore, loco-regional treatments using monoclonal antibodies (Abs) against CD71, offer the best setting for Abs-guided drug therapy, bypass any unspecific toxicity of healthy organs. In a phase I clinical trial by Laske, D.W. et al., anti-CD71 IgG1 linked to the enzymatic moiety of Ricin A chain (RTA) was locally injected to treat leptomeningeal neoplasia. In this setting, only tumor cells expressed adequate levels of CD71 and suffered from cytocidal effects of the drug [6].

In 2010, CD71 was identified as the receptor for human ferritin H-chain (HFt) [7]. HFt shows slightly less binding affinity than Tf for CD71 reducing unspecific binding to ubiquitous CD71 in normal cells. HFt binds to a region of the receptor different from its cognate ligand while retaining its receptor-mediated endosomal internalization. Thus, a new line of research has lately emerged using native or engineered HFt as a nano-vehicle system for drug delivery to tumor masses [8]. Very recently, we have synthesized an HFt derivative, so called HFt-MP-PAS. The outer shield, PAS, is a repetitive polypeptide of Proline-Alanine-Serine sequence and acts as a protective masking moiety [9]. Between PAS and the HFt subunit, a metalloprotease (MP) recognition site is inserted that can be specifically cleaved by tumor proteases. This restricts the selective unmasking of the nano-vehicle and the restoration of the HFt binding capability in the tumor microenvironment. This construct displayed low affinity to CD71 during blood circulation while increasing its serum half-life and facilitating the loading of Doxorubicin (DOXO), an anthracycline chemotherapy drug, at the tumor site [10,11,12].

Interestingly, a recombinant PAS sequence, PASE, formed by the insertion of two glutamic acid residues allowed us to encapsulate another type of chemotherapeutic drug, Mitoxantrone (MIT), at high yields [13]. MIT is an anthracenedione currently used for the management of both haematological and solid tumors, such as Hodgkin lymphoma, non-lymphocytic leukemia, prostate cancer, metastatic breast cancer and relapsed hepatocellular carcinoma ([14,15] pp. 289–315).

As other anthracyclines and anthracenediones based treatments, MIT can lead to serious side effects. Therefore, new formulations are needed to improve the efficacy and tolerability of these drugs.

In a previous paper we demonstrated that targeting PC with HFt-MP-PASE-MIT increased by ten-fold the biodistribution of loaded-MIT at the tumor site compared to its free counterparts [13].

Here, we report for the first time the in vivo efficacy of the new HFt-MP-PASE-MIT nanosystem in a subcutaneous mouse model of PC. The results show that the treatment with HFt-MP-PASE-MIT significantly decreases tumor growth gaining longer animal survivals in comparison with the free drug. All these improvements were obtained without any remarkable side effects on the mice organs.

## 2. Materials and Methods

### 2.1. HFt-MP-PASE-MIT Production

To effectively encapsulate the anti-cancer drug MIT (MedKoo Biosciences, Morrisville, NC, USA), we used the variant form of HFt (named HFt-MP-PASE) recently developed by our group. Protocols for HFt-MP-PASE production and MIT encapsulation (HFt-MP-PASE-MIT) are reported in [13].

### 2.2. The In Vitro Viability XTT Assays and Kinetic Cell Intoxication

Human pancreatic cancer cells, PaCa44, were grown in Dulbecco’s Modified Eagle Medium supplemented with 2 mM glutamine, 10% of FBS, and antibiotics. All cell culture reagents were purchased from Sigma-Aldrich, St Louis, MO, USA. Around 5000 cells were seeded in 90 µL of complete medium in 96-well culture microplates. After 24 h cells were incubated in triplicate with 10 µL of serially diluted Gemcitabine (GEM) (Sigma-Aldrich, St Louis, MO, USA), free MIT, or HFt-MP-PASE-MIT. After 48 h of incubation at 37 °C with drugs, the medium was replaced with fresh medium w/o phenol red supplemented with XTT (2,3-Bis-(2-Methoxy-4-Nitro-5-Sulfophenyl)-2H-Tetrazolium-5-Carboxanilide) reagent (Sigma-Aldrich, St Louis, MO, USA), according to the manufacturer’s instructions. Finally, after a variable time ranging from 1 to 3 h of incubation at 37 °C, cell viability was measured at 450 nm by a microplate reader (VERSAmax, Molecular Devices, Sunnyvale, CA, USA). The percentage of cell viability was estimated by comparing treated cells to mock ones. To compare the killing efficacy, we evaluated the IC50, i.e., the inhibitory compound concentration yielding 50% cell viability.

Kinetic intoxication viability assays were performed with the same procedures described above. Only varied drug concentrations used in the assays (i.e., 1 µM and 0.1 µM of MIT for both loaded-MIT and free MIT) and the choice to analyze the killing efficacy at different time points (i.e., 24, 48, 72 and 96 h of treatment).

### 2.3. Apoptosis Evaluation by Flow Cytometry

In a 24-well plate, almost 150,000 PaCa44 cells were cultured for one day prior to their incubation for 48 h with 0.5 µM of either with HFt-loaded MIT or free MIT drug forms. As control, GEM, a drug currently applied in PC therapy was used at a concentration of 15.2 mM (i.e., 4 mg/mL). At the end of the incubation after some washing steps, cells were detached and apoptosis/necrosis were detected using the Annexin V-FITC labelled assay (ABCAM, Cambridge, UK). Fluorescence was acquired by a BDFACSCanto TM II (Becton Dickinson, San Jose, CA, USA) flow cytometer and analyzed with the FACSDiva Software (Becton Dickinson, San Jose, CA, USA).

### 2.4. Drug Uptake by Tumor Cells

In a 24-well plate, 250,000 PaCa44 cells were cultured for one day prior to their treatment with 0.5 µM of HFt-loaded MIT or free MIT drugs for different time points (i.e., 20, 40, 60, 90, 120 min). MIT cellular uptake was evaluated after thoroughly washing the plates, detaching the cells, and exploiting the MIT fluorescence in the APC channel using a BD FACSCanto TM II flow cytometer. The differential percentage of cellular uptake of 0.5 µM HFt-MP-PASE-MIT versus MIT was calculated following the formula

$$\%\;of\;cellular\;uptake=100\times \frac{\left({MFI}_{\;\mathrm{HFt}-\mathrm{MP}-\mathrm{PASE}-\mathrm{MIT}\;}-{MFI}_{\;\mathrm{CTRL}}\right)}{\left({MFI}_{\;\mathrm{MIT}\;}-{MFI}_{\;\mathrm{CTRL}}\right)}\;\mathrm{MFI}=\mathrm{Mean}\;\mathrm{Fluorescence}\;\mathrm{Intensity}$$

### 2.5. In Vivo Therapeutic Evaluation

For the subcutaneous model, 5-week-old female CD1 nude mice (Charles River Laboratories; Calco, LC, Italy) were injected subcutaneously in the right flank with 4 × 10^6^ PaCa44 cells resuspended in 200 μL of PBS. When subcutaneous tumor reached a volume of about 80–100 mm^3^, mice were randomized in groups of four and intravenously injected in the tail vein with saline, MIT, or HFt-MP-PASE-MIT. The treatment dose of 1.4 mg/Kg was normalized to MIT concentration and mice were treated twice a week for three consecutive weeks for a total of 6 injections. Tumor volume was measured with a caliper and mouse weight was monitored. Moreover, mice were observed for signs of distress or pain twice a week for all the duration of the experiment. A tumor volume ≥1500 mm^3^ was chosen as endpoint after which mice were sacrificed. Overall survival rate was also evaluated. Animal studies were performed according to a protocol approved (12 June 2014) by the Institutional Animal Care and Use Committee at University of Verona and authorized by the Italian Ministry of Health (Protocols no. 128/2014-B); in compliance with the principles of the European Community Council Directives.

### 2.6. Histological Evaluation of Primary Tumors and Other Organs

The last stage of our experimental study entailed the sacrifice of tumor-bearing mice by a CO_2_ overdose via facial mask. Consequently, primary tumors and various organs, such as liver, lungs, kidneys and spleen were excised and preserved for analysis. Preservation was mediated via a fixation step with 4% buffered formalin for 4 h, dehydration in ethanol gradient (from 70% to 100%), followed by two passages in 100% xylene. Then samples were paraffin-embedded and cut with a microtome to obtain sections of 5 µm thickness. For the histological evaluation, the slices were stained with hematoxylin/eosin (Bioptica, Milan, Italy) and examined with light microscopy using an Olympus BX-51 microscope (Olympus Italy, Segrate, MI, Italy), equipped with a KY-F58 CCD camera (Nikon Italy, Campi Bisenzio, FI, Italy).

### 2.7. Statistical Analysis

Experimental data were expressed as a mean ± SEM and the statistical significance was calculated using a Sample T test. A *p* < 0.05 value was considered statistically significant. Survival percentages were estimated using Kaplan-Meier methods and survival curves were compared using the log-rank test.

## 3. Results

### 3.1. In Vitro Cytotoxicity on PaCa44 Cells

**Figure 1:**
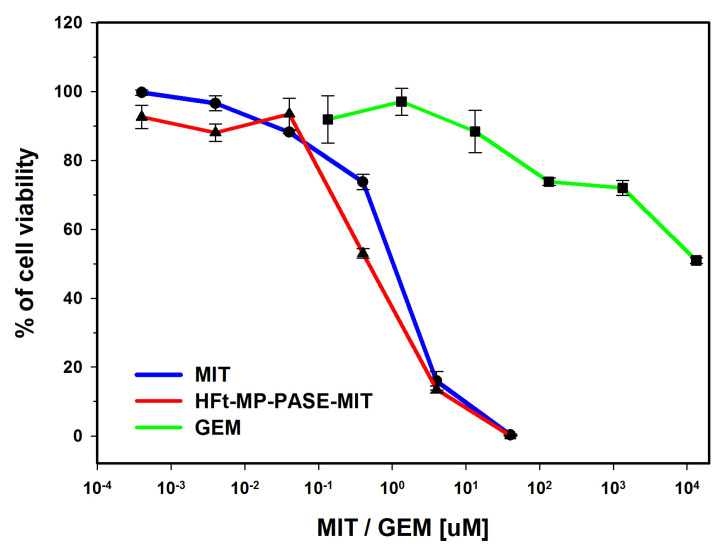
Cytotoxic potential of free or loaded-MIT using XTT assay. PaCa44 pancreatic tumor cells were treated 48 h with serial dilution of HFt-MP-PASE-MIT, MIT or GEM. The percentage of cell viability was measured by XTT assay, and means ± SEM were calculated from three independent experiments.

Our previous findings demonstrated that in vitro HFt-MP-PASE-MIT nanocages are efficient in killing several cancer cell lines as efficiently as free drug form [13]. To confirm our observation, we first aimed in this study to test the cytotoxic capability of our generated HFt-MP-PASE-MIT on PaCa44 cell line given that this cell line was also used in our in vivo experiments. We evaluated the potency of our nano-formulation using XTT assay while collecting the data from three different experiments. As depicted in Figure 1, PaCa44 cells exposed to HFt-MP-PASE-MIT or free MIT for 48 h showed quite similar drug sensitivity with an IC50 drug response of 0.47 ± 0.07 µM and 1 ± 0.12 µM for loaded and free MIT concentrations, respectively. It is noteworthy that GEM, a drug currently used in PC therapy, showed in the same assay, a considerably lower drug killing efficacy with IC50 = 13.5 ± 1.25 mM.

### 3.2. Flow Cytometric Analysis of Necrosis or Apoptosis

In order to identify key cellular markers of ‘cell death’ or apoptosis, we have measured the expression level of phosphatidylserine using flow cytometry. The Annexin V-FITC kit was employed with propidium iodide (PI) to differentiate apoptotic from necrotic cells. Annexin V has high binding affinity for phosphatidylserine proteins translocated from the inner side of the plasma membrane to its outer leaflet; while necrotic cells lost their cellular integrity and thereby are permeable to PI dye that in turn intercalates with the nuclear DNA and is visible by red fluorescence. We treated PaCa44 cells with 0.5 µM of our nano-formulation or free MIT for 48 h; GEM (4 mg/mL) that is equivalent to 15.2 mM was used as control. Results of Annexin V binding and PI uptake were quantified and summarized in Table 1. HFt-MP-PASE-MIT and MIT showed quite similar levels of early apoptosis 11.30% ± 1.00 and 13.25% ± 1.85, respectively. Also, the amount of cells in late apoptosis was quite similar reaching 4.95% ± 0.45 and 6.35% ± 1.05 for encapsulated and MIT-free form, respectively. Moreover, the level of cell death for necrosis was superimposable. GEM showed a similar percentage of cells in early and late apoptosis 10.01% ± 0.20 and 7.60% ± 0.50, but at a drug concentration of 15.2 mM, that is about 4 Log higher with respect to MIT concentration.

### 3.3. Kinetics of Cell Intoxication

**Figure 2:**
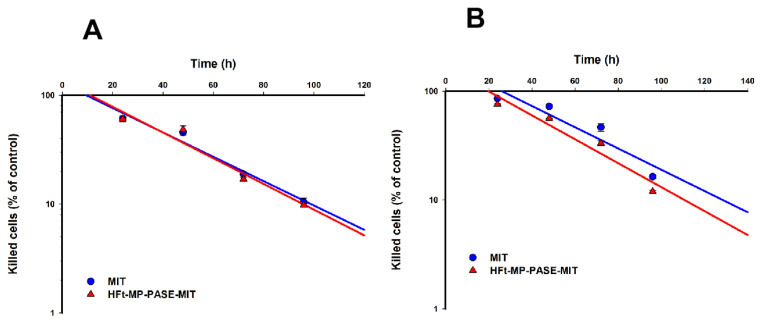
Kinetic curves of cell killing by HFt-MP-PASE-MIT and MIT. PaCa44 pancreatic tumor cells were treated for different time points with an equivalent MIT dose of 1 µM (**A**) or 0.1 uM (**B**) and viability was assessed by XTT assay. Linear regression was applied to fit the experimental data ((**A**), r^2^ = 0.97 and r^2^ = 0.95 for MIT and HFt-MP-PASE-MIT, respectively; (**B**) r^2^ = 0.88 and r^2^ = 0.93 for MIT and HFt-MP-PASE-MIT, respectively). Three independent experiments were performed ± SEM.

A previous report by our group demonstrated that HFt-MP-PASE-MIT is rapidly internalized by CD71 and traffic intracellularly via the endosomal pathway. Then, loaded-MIT escapes from these organelles and arrives at the nuclear level, where MIT produces DNA breaks and cross-links. Instead, free MIT enters via passive diffusion across the plasma membrane and its distribution is then between nucleus and cytoplasm [13]. The rate of drug uptake can directly reflect the kinetics of tumor cell killing and is pivotal in studying the efficacy of chemotherapeutic agents. In order to examine the kinetics of tumor cell death, PaCa44 cells were treated with HFt-MP-PASE-MIT or MIT alone, and subjected to XTT assays at different time points. Using an MIT normalized dose of 1 µM we observed that HFt-MP-PASE-MIT killed 1 Log of tumor cells (i.e., 90% of tumor cells) in 96 h. MIT eliminated the same amount of cells in a quite similar time of 100 h (Figure 2A). When a ten-fold dilution of the drug was used, 0.1 µM, a slight difference was noticeable in the kinetic killing curves. HFt-MP-PASE-MIT killed 90% of tumor cells in 110 h while MIT cytotoxicity showed same killing rate at 128 h (Figure 2B). Moreover, the lag phase, the time elapsing before cell death, was quite overlapping between the two drugs (i.e., 10 h for MIT and 10.5 h for HFt-MP-PASE-MIT). When the same parameter was analyzed in the killing kinetic curves obtained after treatments with a MIT concentration of 0.1 µM, HFt-MP-PASE-MIT showed a shorter lag phase compared to its free counterpart (20 h and 27 h, respectively).

### 3.4. Uptake in Tumor Cells

Given the comparable curves in the cell killing kinetics for both MIT forms, it was challenging to examine the rate of drug uptake especially given that encapsulated MIT molecules possess larger size and possibly different charge. To tackle our concern, we set to study the uptake of 0.5 µM of drugs by PaCa44 cells at 37 °C at different time points (i.e., 20-40-60-90-120 min). It is worth noting that the fluorescent emission of MIT can be captured in the APC channel using flow cytometry. As summarized in Table 2, at all time points, HFt-MP-PASE-MIT demonstrated a superior uptake into the cells with more MIT (about 35%) accumulated in the target cells when the drug is loaded on the nanosystem.

### 3.5. Therapeutic Efficacy In Vivo

The in vivo efficacy of our nanosystem was assessed in a subcutaneous PC mouse model. PaCa44 cells (4 × 10^6^) were injected in the left flank of 5-week-old female CD1 NUDE mice (*n* = 4). After 16 days, when established tumors had gained a volume of about 80–100 mm^3^, mice were randomized and assigned at one of the four groups (i.e., saline, free drug and encapsulated drug). Treatments were performed twice a week for three consecutive weeks by intravenous injection in the tail vein. The treatment doses were normalized to MIT dose (i.e., 1.4 mg/kg). The dose was established considering a Maximum Tolerated Dose (MTD) for MIT of 9 µmol/kg (i.e., about 4 mg/kg). So, we used about 1/3 of the MTD at each treatment for a total amount of about 8 mg that is the double of the MTD.

**Figure 3:**
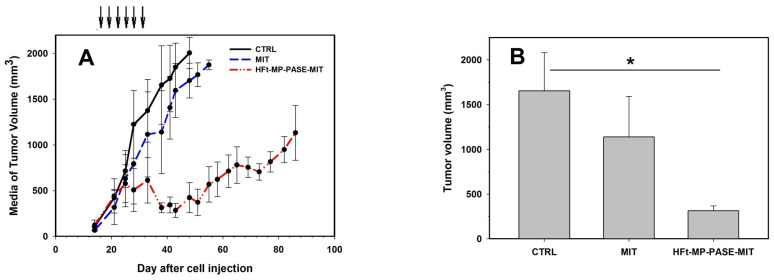
(**A**) Growth curves representing the media of the tumor volume for each of the treated groups: HFt-MP-PASE-MIT, MIT or saline (CTRL, control); *n* = 4. Arrows indicate the six days of treatment administration. (**B**) Tumor volume of the three different groups measured at day 38 after tumor cell injection. * *p* < 0.05.

Tumors in control mice grew progressively, showing a tumor doubling time of about 5 days during the exponential growth phase (Figure 3A). Only a very limited growth delay was observed in the group treated with MIT alone, but without any statistical significance. In contrast, we observed a high difference in tumor growth when mice were treated with HFt-MP-PASE-MIT. After an initial superimposable growth of tumor compared to mock treated animals, tumor volume started to significantly decrease when the 4th dose was administered.

One week post-treatment (i.e., day 38), there was a significant difference between tumor volume of control (V = 1653.8 ± 428.5 mm^3^) and HFt-MP-PASE-MIT treated mice (V = 313.2 ± 54.6 mm^3^, *p* = 0.019) (Figure 3B). At day 48, when the last mouse of the control group was sacrificed, the media of tumor volume between MIT (V = 1703.1 ± 189.2 mm^3^) and HFt-MP-PASE-MIT (V = 423.6 ± 164.4 mm^3^) groups varied significantly (*p* = 0.0075), (Figure S1).

**Figure 4:**
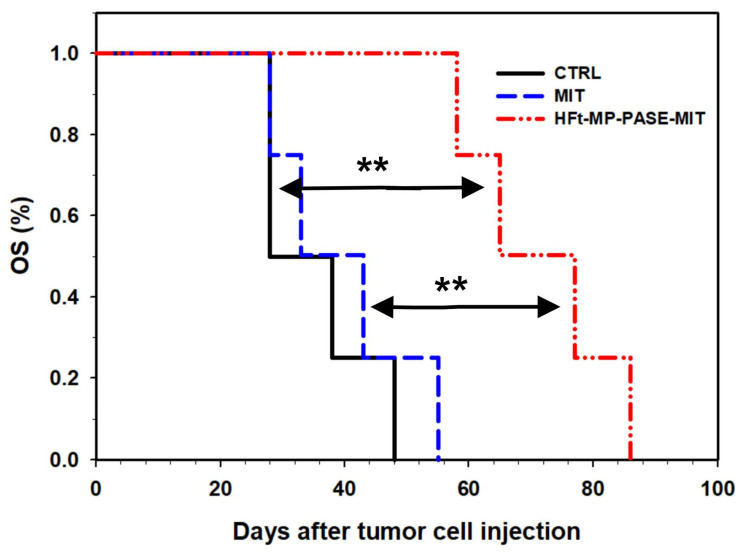
Kaplan-Meier survival plot. Mice with established s.c. tumor of PaCa44 cells were treated twice a week for three consecutive weeks with HFt-MP-PASE-MIT, MIT or saline (CTRL, control). (*n* = 4) ** *p* < 0.01.

Using the Kaplan-Maier plot, mice treated with 0.5 µM of HFt-MP-PASE-MIT achieved a median survival time (OS50%) of 65 days, which is considerably greater than OS50% of 28 days and 33 days of control mice and MIT groups, respectively. Moreover, it is important to highlight the statistically significance of this increased survival (i.e., data of the Log Rank Test, HFt-MP-PASE-MIT vs. MIT *p* = 0.006, HFt-MP-PASE-MIT vs. control *p* = 0.006 and MIT vs. control *p* = 0.531) (Figure 4). Based on the *p* values, untreated and MIT treated groups did not show significant improvement in the survival of mice. Whereas, encapsulated-MIT exhibited remarkable prolonged survival periods of almost additional 33 days compared to MIT treated groups.

Furthermore, it is important to underline that some of the mice treated with HFt-MP-PASE-MIT (i.e., 2/4, 50%) were sacrificed before reaching a tumor volume ≥1500 mm^3^, our abortion criteria, because the high cytotoxic activity of the drug on the tumor had created eschars (Figure S2). Therefore these mice were sacrificed to not create excessive pain in mice.

Regarding the nonspecific toxicity of our therapy, we observed that HFt-MP-PASE-MIT mice experienced a maximal weight loss of about 10% during the treatments, but then their weight was restored back to normal value. (Figure S3) Otherwise, MIT treated mice demonstrated less capability to regain their original weight. In the same setting, control mice showed an increment in their weight of no more the 5%.

### 3.6. Histological Evaluation of Primary Tumor Mass

**Figure 5:**
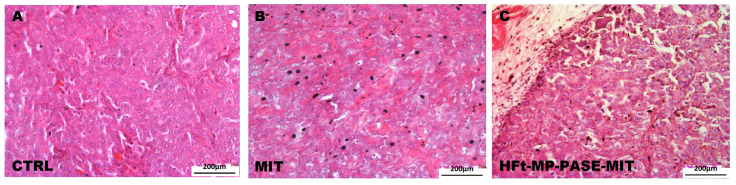
Histology of primary tumor mass. Images show the parenchyma of primary tumors in all the experimental groups. (**A**) A representative image of primary tumor mass collected from the control group treated with saline; (**B**) mice treated with free MIT; (**C**) mice treated with HFt-MP-PASE-MIT.

Histological evaluation of parenchyma primary tumor mass was performed using hematoxylin and eosin stain to verify the treatment efficacy of HFt-MP-PASE-MIT nanosystem. In fact as depicted in Figure 5A, tumor explanted from a representative mouse belonging to the control group showed very dense parenchyma morphology typical of pancreatic tumors with the presence of numerous capillaries. While the representative tumor, explanted from mouse belonging to the group treated with HFt-MP-PASE-MIT, showed numerous necrosis areas and scarce vascularization (Figure 5C). The treatment with free MIT, representing a non-targeted drug, is less efficient in reducing primary tumor viability (Figure 5B). Indeed, MIT administration showed a dense structure of primary tumor with rare areas of necrosis and a good presence of capillaries. Similar histological tumor mass profiles were screened for each group treatment and the selected images display representative morphologies.

### 3.7. Histological Evaluation of Other Organs

Histological evaluation of lungs, kidneys, spleen, liver and heart specimens was performed for all the treated mice, in order to inspect any organ structural abnormalities and toxicity effects.

**Figure 6:**
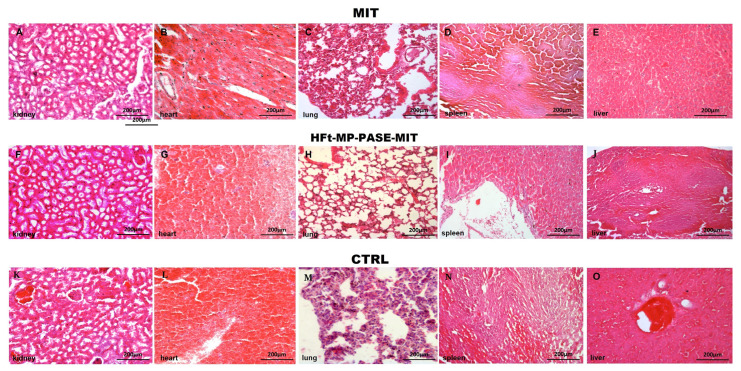
Histological evaluation of the integrity of the organs architecture. Several tissues as kidneys, hearth, lungs, spleen and liver were explanted from mice at sacrifice. The panel shows light microscope images from mice treated with MIT (images **A**–**E**), HFt-MP-PASE-MIT (images **F**–**J**) or saline (images **K**–**O**).

Histology of different organ tissues, reported in Figure 6, showed that both MIT and HFt-MP-PASE-MIT did not exert any toxic effects on the overall architecture and organization of cells. The selected images portray representative profiles of other tissue sections; thus, reflecting the overall preserved structure of various organs unaffected by the type of applied drugs.

## 4. Discussion

PC represents one of the most complex challenge of modern oncology. To date, significant progresses have been made in the field of surgery especially in the case of localized tumors in which this treatment can be curative (roughly 30% 5 year relative survival) [16]. Instead, in the treatment of relapsing or already metastatic forms, the results have been relatively modest in term of survival despite the considerable efforts made in the study of the PC pathology and in the design of new drugs and therapeutic protocols. Therefore it remains a pressing issue to test newly engineered drugs in validated in vitro and in vivo assays for quicker and better clinical advances.

Currently, scientists address this issue by modifying existing drugs for enhanced biodistribution at the tumor site, extended half-life in the serum, and increased penetration into cancer cells, while overcoming the resistance mechanisms that often arise during therapy [17].

In our case, we used recently engineered nanosystems of the human HFt that can be loaded with different chemotherapeutic agents, increasing their plasma half-life, shielding them from enzymes present in the blood and allowing greater accumulation at the tumor level [18].

This increased tumor-specificity is mainly due to the ferritin H-type capability to bind a specific receptor, the TfR1 (CD71), widely overexpressed in different tumor histotypes [7]. The overexpression of CD71 also correlates with a poor prognosis in some solid tumors such as breast cancer [19], hepatocellular carcinoma [20], cholangiocarcinoma [21], renal cell carcinoma [22] and pancreatic cancer [23]. The tumorigenicity associated with the expression of TfR is not only due to the greater demand for iron, an essential cofactor for the functionality of many enzymes including those involved in the synthesis of nucleic acids feeding cellular proliferation, but also to other factors [24,25]. In fact, some studies have highlighted the involvement of TfR in other mechanisms sustaining tumor growth, such as NFkB activation and the increased ROS production [26].

For targeted PC chemotherapy, we decided to trap the drug MIT in the internal cavity of our novel HFt-MP-PASE nanosystem, where the introduction of a masking polypeptide (PASE), cleavable at the specific MP site by the tumor metalloprotease allowed us to improve the specific targeting [13]. In the past, MIT has showed a high cytotoxic activity on a wide range of tumor cell lines and a higher killing activity with respect to GEM, a standard care in PC therapy, as demonstrated by in vitro assays [27].

This paper represents the first comprehensive study on the anti-tumor activity of HFt-MP-PASE-MIT providing in vitro and in vivo evidence of its killing capability.

In the past decade, other groups attempted similar strategies. Janko et al. loaded MIT on iron oxide nanoparticles and evaluated their killing activity on a spheroid model of HT-29 human colon cancer cells [28]. In this model, MIT showed superior capability to reduce spheroids regrowth than the MIT loaded nanoparticles. Moreover, Keese group applied in vivo biocompatible beads loaded with MIT (i.e., drug eluting beads) to treat pancreatic peritoneal carcinomatosis in mice [29]. They showed that local treatment by peritoneal injection was able to decrease the deaths associated with high dosage of free MIT treatments. Therefore, demonstrating that MIT-loaded nanosystem can decrease the non-specific toxicity of this drug.

Regarding MIT in vivo efficacy, Song et al. loaded MIT on liposomes derivatized with the peptide gonadorelin to target the receptor of LHRH (Luteinizing Hormone Releasing Hormone) that is highly expressed in tumors [30]. These LHRH-MIT-LIPs demonstrated a decrease in MCF-7 subcutaneous tumor in mice when administered at 2.5 mg/kg every week for three weeks. In many studies conceived to increase the MIT efficacy liposomes of about 100 nm were utilized as nanovectors due to their easy production, batch to batch reproducibility, biocompatibility and biodegradability. It is noteworthy that PC microenvironment shows a high level of fibrotic processes mainly attributed to the Cancer-Associated Fibroblasts (CAFs). Resident CAFs are also activated to produce extracellular matrix components as collagen, fibronectin and hyaluronic acid [31]. Cargo-nanovectors with reduced size could prove advantageous to diffuse in this unfavourable environment with increased stiffness, hydrostatic pressure, and hypovascularization. Remarkably, our engineered HFt-MP-PASE has an external diameter ranging between 12 and 18 nm, way smaller than liposome diameter, marking them as suitable nanocarriers for PC therapy.

The in vitro characterization of our nano-formulation demonstrated an excellent cytotoxic activity in the micromolar range at 48 h, comparable to that of the free MIT and much greater than that of GEM. Moreover, encapsulated MIT, just like their free MIT forms, showed similar cell-cycle inhibition inducing both apoptosis and necrosis at comparable rates after 48 h of administration. As we observed from the XTT assay and apoptosis tests, GEM demonstrated comparable levels of cell death in both apoptosis and necrosis but at a much greater drug concentration of about 4 Log higher. Thus, our MIT-nanocarriers can penetrate the cells reaching nuclear DNA and causing cell death [13].

Moreover, studying the intoxication kinetics, the cytocidal effect of HFt-MP-PASE-MIT was also analyzed. In spite of a more complex internalization step and routing inside cells, HFt-MP-PASE-MIT showed a superimposable rate of tumor cell killing at an equivalent dose of 1 µM in MIT. Surprisingly, at a ten-fold diluted concentration, the kinetic of tumor cell killing was faster with respect to MIT alone. When we moved to study the in vivo efficacy on stabilization/retardation of established tumor growth in s.c. mouse models of PC, we observed an intriguing delay on tumor regrowth.

Furthermore, we appreciated a statistically significant reduction of tumor volume of 81.1% between HFt-MP-PASE-MIT and mock treated mice at day 38 (*p* < 0.05, Figure 3b). Ten day after, at the day 48, also the reduction of tumor volume between HFt-MP-PASE-MIT and MIT (volume reduction of 75.1%) became statistically significant (*p* < 0.01, Figure S1). The high cytotoxic activity and the loaded-drug accumulation at the tumor mass was confirmed in 2 of 4 HFt-MP-PASE-MIT treated mice by the formation of large eschars on the flank where tumor was established (Figure S2). These eschars forced us to suppress these 2 mice before reaching our abortion criteria and therefore the survival time of HFt-MP-PASE-MIT treated group was underestimated.

A possible hypothesis to explain this notable difference in the in vivo efficacy of encapsulated MIT compared to free MIT, as well as a better tumor biodistribution [13], could be an increase in TfR1 expression on tumor cells due to hypoxic condition with HIF-1 increase [32].

Regarding the non-specific toxicity and side effects of MIT administration, histological analysis of various tissue organs clearly showed that both MIT and HFt-MP-PASE-MIT do not modify the architecture and cellular organization of the analyzed organs.

## 5. Conclusions

Our study presents promising results from in vitro and in vivo applications of MIT drug loaded on HFt modified nanocages as effective means to control the growth of PC cells. Compared to freely unloaded MIT molecules, HFt-MP-PASE-MIT demonstrated a superimposable capability to kill and intoxicate tumor cells despite its large size and mode of entry using a receptor (CD71)-mediated uptake process. Complementary data from in vivo experiments clearly demonstrated that when MIT is delivered to tumor mass by HFt nanocage, the growth of subcutaneous induced-tumor is much slower, thereby prolonging the overall survival rate of treated mice. Our findings demonstrate an in vivo efficacy of our MIT variant that is superior to any other tested forms. All this is achieved without compromising the overall health status of the mice and the structure of various body organs. In conclusion, HFt-MP-PASE-MIT offers reliable and favorable nanoplatforms that could be also effective in delivering various drugs to advance PC cancer therapy.

## 6. Patents

P.C. and E.F. are inventors of patent application EP3186192B1 held by Thena Biotech that covers fusion proteins based on human ferritins and methods of use thereof.

## Figures and Tables

**Table 1:** PaCa44 cell apoptosis detected by flow cytometry after 48 h of treatment.

Drug Concentration	Early Apoptosis	Late Apoptosis	Viable Cells	Necrosis
GEM 15.2 mM	10.01% ± 0.20	7.60% ± 0.50	58.00% ± 2.60	24.25% ± 2.25
MIT 0.5 µM	13.25% ± 1.85	6.35% ± 1.05	56.65% ± 1.45	23.80% ± 0.60
HFt-MP-PASE-MIT 0.5 µM	11.30% ± 1.00	4.95% ± 0.45	60.10% ± 4.90	23.70% ± 3.50

**Table 2:** Percentage of the differential cellular uptake of 0.5 µM HFt-MP-PASE-MIT versus MIT at 37 °C detected by flow cytometry at different time points.

	20 min	40 min	60 min	90 min	120 min
HFt-MP-PASE-MIT vs. MIT	133.07% ± 7.21	133.25% ± 7.37	139.49% ± 6.71	142.02% ± 7.00	138.37% ± 0.74

## Data Availability

All data are available in the main text or the Supplementary Materials.

## Supplementary Materials

The following are available online at https://www.mdpi.com/article/10.3390/biomedicines9111622/s1, Figure S1: Tumor volume of the three group of treated mice (HFt-MP-PASE-MIT, MIT or saline) at day 48 after tumor cell injection. (*n* = 4) * *p* < 0.05, ** *p* < 0.01; Figure S2: Pictures of the tumors of HFt-MP-PASE-MIT treated mice at sacrifice. Mice 300 and 304 were sacrificed before they gain a tumor volume ≥1500 mm^3^ due the high eschars formed for necrosis of tumor cells. Mouse 302, included in the same treatment group, did not developed eschars; Figure S3: Percentage of mouse weight change during the treatment with HFt-MP-PASE-MIT, MIT or saline. (*n* = 4).
