# Supplementary material for: Mitoxantrone-Loaded Nanoferritin Slows Tumor Growth and Improves the Overall Survival Rate in a Subcutaneous Pancreatic Cancer Mouse Model
Source: Biomedicines. 2021 Nov 5;9(11):1622. doi: 10.3390/biomedicines9111622 (PMC8615572; doi:10.3390/biomedicines9111622)
Supplement: Supplementary file 1 [file biomedicines-09-01622-s001.zip › biomedicines-1416574-supplementary.pdf]

Supplementary Information

Mitoxantrone-Loaded Nanoferritin Slows Tumor Growth and Improves the Overall Survival Rate in a Subcutaneous Pancreatic Cancer Mouse Model

Giamaica Conti [1,†], Martina Pitea [2,3,†], Riccardo Ossanna [1], Roberta Opri [4], Giada Tisci [2], Elisabetta Falvo [5], Giulio Innamorati [6], Esther Ghanem [7], Andrea Sbarbati [1,‡], Pierpaolo Ceci [5,*,‡] and Giulio Fracasso [4,*,‡]

1 Department of Neurological and Movement Sciences, University of Verona, 37134 Verona, Italy; giamaica.conti@univr.it (G.C.); riccardo.ossanna@univr.it (R.O.); andrea.sbarbati@univr.it (A.S.)
2 Department of Biochemical Sciences, University Sapienza, 00185 Rome, Italy; martina.pitea@uniroma1.it (M.P.); tisci.1887341@studenti.uniroma1.it (G.T.)
3 Center for Life Nano Science@Sapienza Istituto Italiano di Tecnologia, 00161 Rome, Italy
4 Department of Medicine, University of Verona, 37134 Verona, Italy; roberta.opri@gmail.com
5 Institute of Molecular Biology and Pathology, CNR—National Research Council of Italy, 00185 Rome, Italy; elisabetta.falvo@cnr.it
6 Department of Surgical Sciences, Dentistry, Gynecology and Pediatrics, Section of Surgery, University of Verona, 37134 Verona, Italy; giulio.innamorati@univr.it
7 Department of Sciences, Notre Dame University-Louaize, XJ28+73C Zouk Mosbeh, Lebanon; eghanem@ndu.edu.lb
* Correspondence: pierpaolo.ceci@cnr.it (P.C.); giulio.fracasso@univr.it (G.F.); Tel.: +39-06-4991-0761 (P.C.); +39-04-5812-6449 (G.F.)
† These authors contributed equally to this work.
‡ These authors share senior authorship.

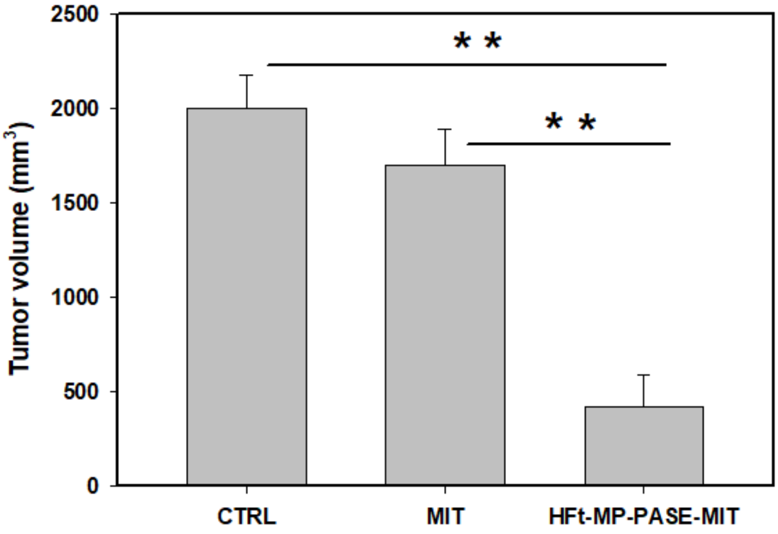

Figure S1. Tumor volume of the three group of treated mice (HFt-MP-PASE-MIT, MIT or saline) at day 48 after tumor cell injection. (n=4) *p<0.05, **p<0.01

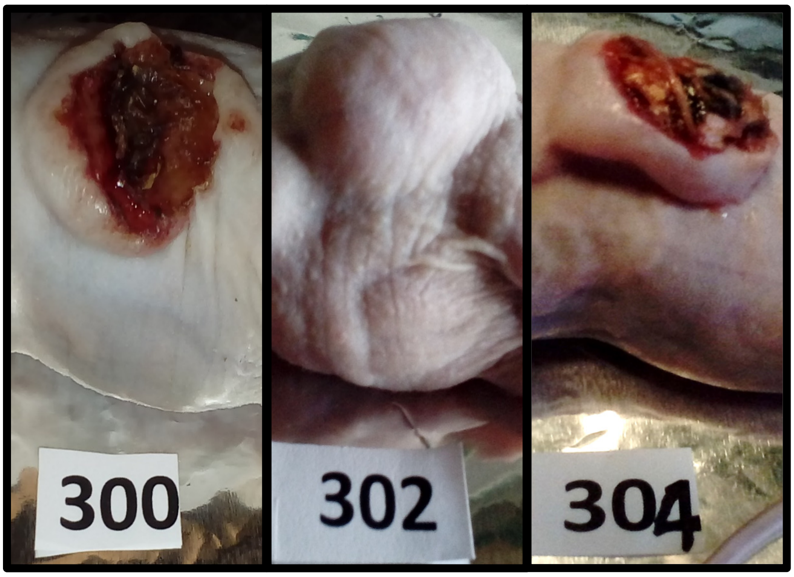

Figure S2. Pictures of the tumors of HFt-MP-PASE-MIT treated mice at sacrifice. Mice 300 and 304 were sacrificed before they gain a tumor volume ≥1,500 mm^3 due the high eschars formed for necrosis of tumor cells. Mouse 302, included in the same treatment group, did not developed eschars.

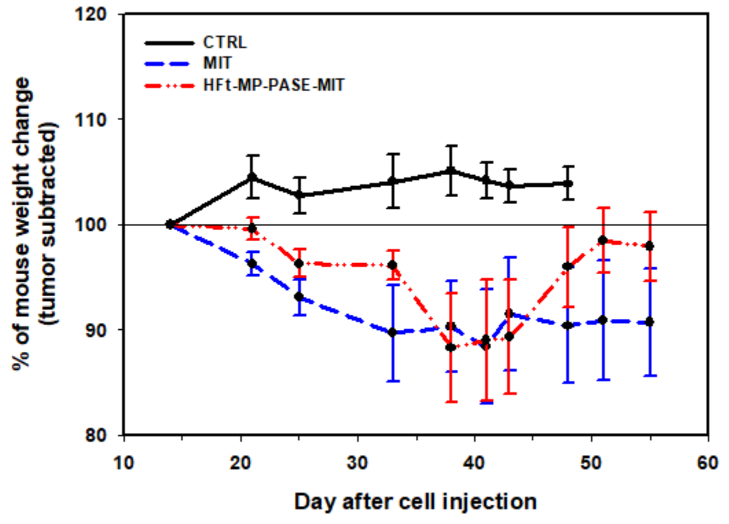

Figure S3. Percentage of mouse weight change during the treatment with HFt-MP-PASE-MIT, MIT or saline. (n=4)
